# Supplementary figures and images for: Reactive Bergmann glia play a central role in spinocerebellar ataxia inflammation via the JNK pathway
Source: J Neuroinflammation. 2023 May 26;20:126. doi: 10.1186/s12974-023-02801-1 (PMC10214658; doi:10.1186/s12974-023-02801-1)

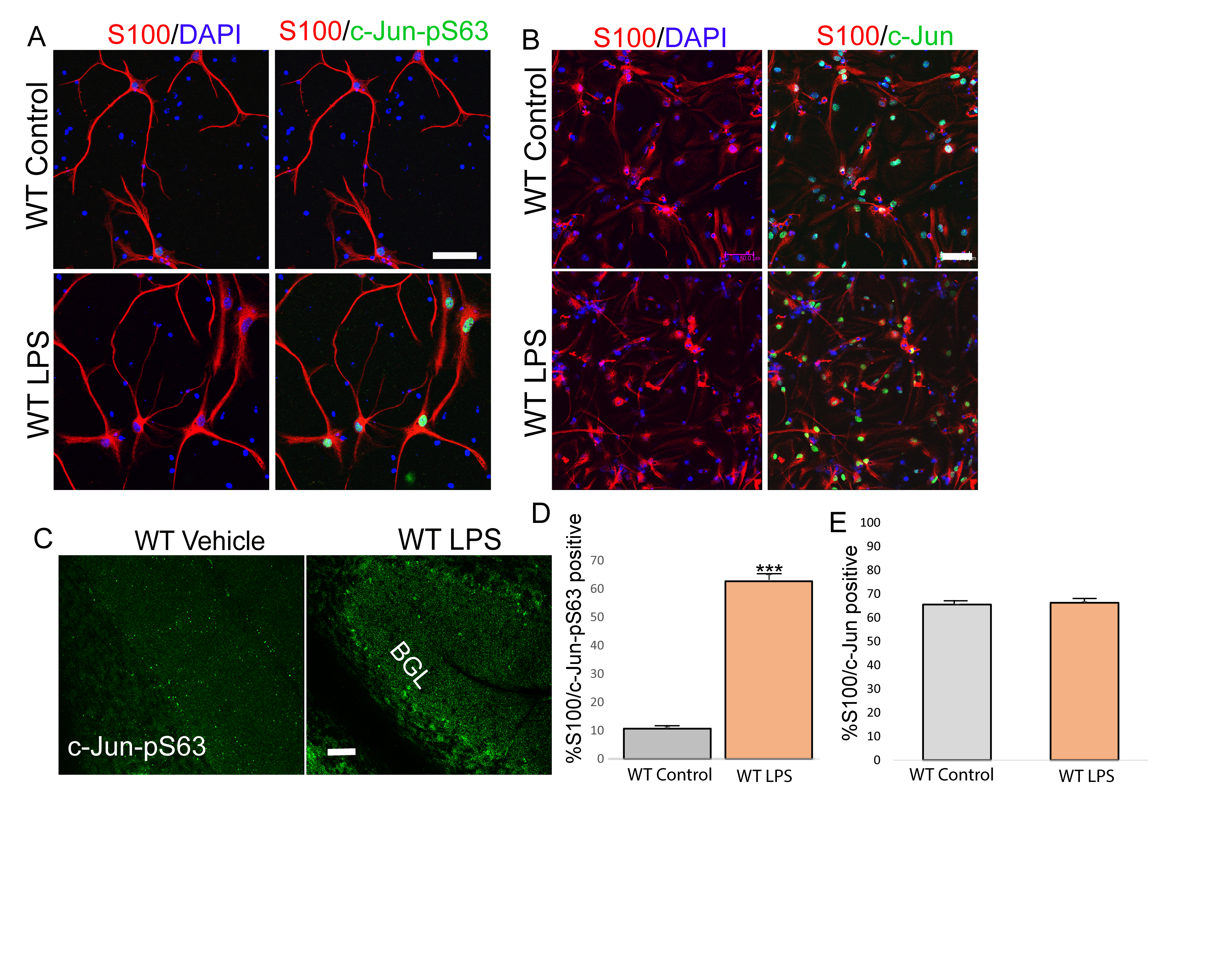

Supplement: Supplementary file 1 — Additional file 1: Figure S1. Lipopolysaccharide induces c-Jun phosphorylation in Bergmann glia in vitro and in vivo. A Immunostaining of S100 with c-Jun-pS63 in DIV6 neuronal/glial cerebellar cultures generated from P4 mice and treated with PBS or LPS. B Immunostaining of S100 with total c-Jun. Slides were stained for nuclei using DAPI that labeled all the cells including glia in this mixed population. Scale bars = 50 μm. C Immunostaining of the cerebellum for c-Jun-pS63 antibody in wild-type mice treated with LPS or vehicle by intraperitoneal injection daily for 5 days. Scale bar = 100 μm. D Quantification of S100/c-Jun-pS63 double-positive cells shown in A. Quantification of S100/c-Jun double-positive cells shown in B. n = 3 individual cultures. ***P < 0.001. [file 12974_2023_2801_MOESM1_ESM.tif]

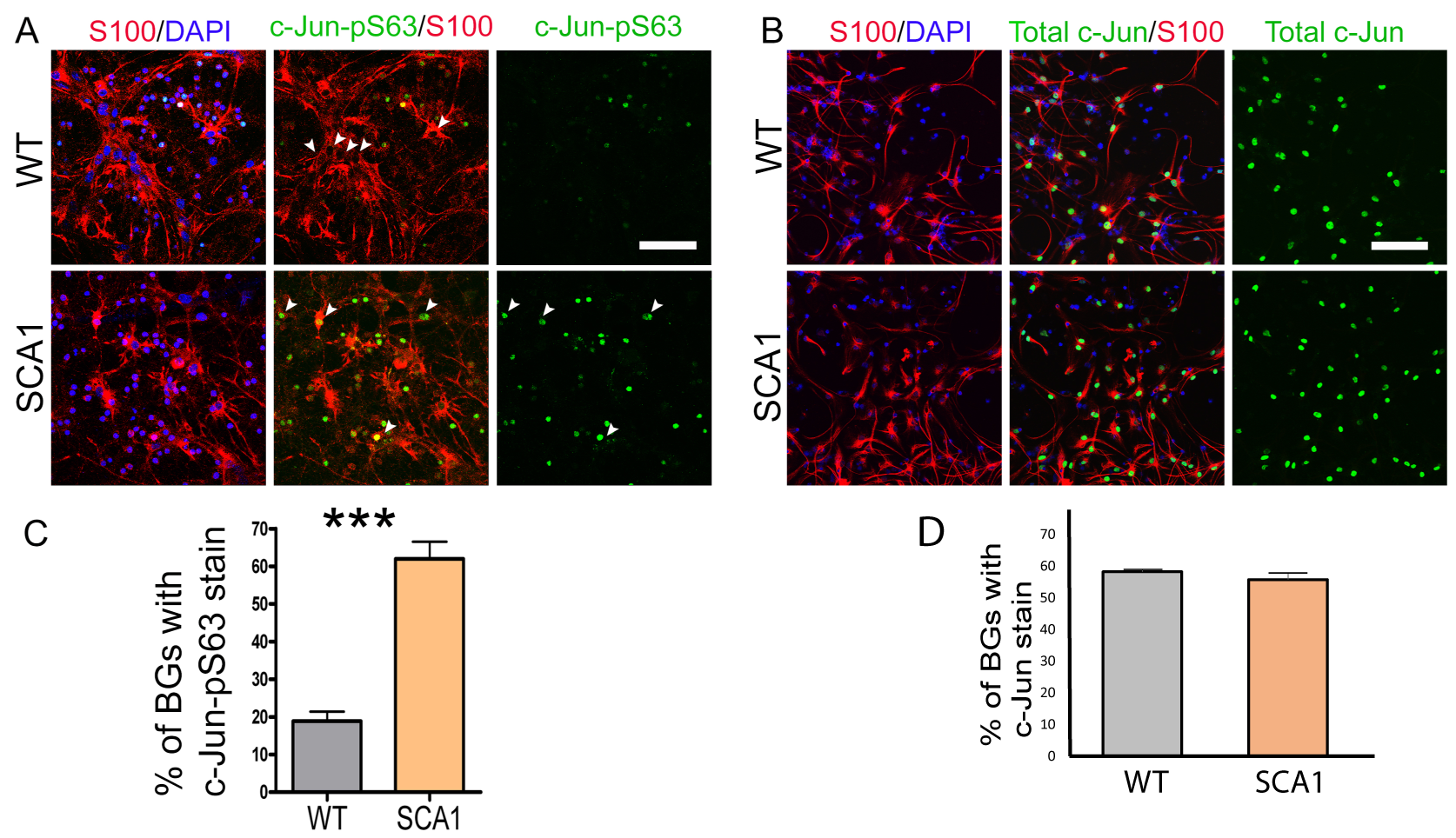

Supplement: Supplementary file 2 — Additional file 2: Figure S2. In vitro isolated Bergmann glial cultures exhibit enhanced c-Jun phosphorylation. A, B DIV6 neuronal/glial cerebellar cultures generated from P4 SCA1 or wild-type mice and immunostained with S100 either with A c-Jun-pS63 or B total c-Jun. White arrowheads indicate examples of S100/c-Jun-pS63 double-positive cells. Scale bar = 100 μm. C Quantification of S100/c-Jun-pS63 double positives shown in A. n = 3 individual cultures. ***P < 0.001. D Quantification of S100/c-Jun double-positive cells shown in B. n = 3 individual cultures. [file 12974_2023_2801_MOESM2_ESM.tif]
